# Novel heterozygous truncating titin variants affecting the A‐band are associated with cardiomyopathy and myopathy/muscular dystrophy

**DOI:** 10.1002/mgg3.1460

**Published:** 2020-08-20

**Authors:** Kelly A. Rich, Tia Moscarello, Carly Siskind, Guy Brock, Christopher A. Tan, Matteo Vatta, Thomas L. Winder, Bakri Elsheikh, Leah Vicini, Brianna Tucker, Marilly Palettas, Ray E. Hershberger, John T. Kissel, Ana Morales, Jennifer Roggenbuck

**Affiliations:** ^1^ The Ohio State University Wexner Medical Center The Ohio State University Columbus OH USA; ^2^ Stanford Center for Inherited Cardiovascular Disease Stanford University Stanford CA USA; ^3^ Stanford Health Care Stanford University Stanford CA USA; ^4^ The Ohio State University Center for Biostatistics The Ohio State University Columbus OH USA; ^5^ Invitae Corporation San Francisco CA USA

**Keywords:** dilated cardiomyopathy, genotype‐phenotype correlation, skeletal myopathy, *TTN*, variant interpretation

## Abstract

**Background:**

Variants in *TTN* are frequently identified in the genetic evaluation of skeletal myopathy or cardiomyopathy. However, due to the high frequency of *TTN* variants in the general population, incomplete penetrance, and limited understanding of the spectrum of disease, interpretation of *TTN* variants is often difficult for laboratories and clinicians. Currently, cardiomyopathy is associated with heterozygous A‐band *TTN* variants, whereas skeletal myopathy is largely associated with homozygous or compound heterozygous *TTN* variants. Recent reports show pathogenic variants in *TTN* may result in a broader phenotypic spectrum than previously recognized.

**Methods:**

Here we report the results of a multisite study that characterized the phenotypes of probands with variants in TTN. We investigated *TTN* genotype‐phenotype correlations in probands with skeletal myopathy and/or cardiomyopathy. Probands with *TTN* truncating variants (*TTN*tv) or pathogenic missense variants were ascertained from two academic medical centers. Variants were identified via clinical genetic testing and reviewed according to the American College of Medical Genetics criteria. Clinical and family history data were documented via retrospective chart review. Family studies were performed for probands with atypical phenotypes.

**Results:**

Forty‐nine probands were identified with *TTN*tv or pathogenic missense variants. Probands were classified by clinical presentation: cardiac (n = 30), skeletal muscle (n = 12), or both (cardioskeletal, n = 7). Within the cardioskeletal group, 5/7 probands had heterozygous *TTN*tv predicted to affect the distal (3’) end of the A‐band. All cardioskeletal probands had onset of proximal‐predominant muscle weakness before diagnosis of cardiovascular disease, five pedigrees support dominant transmission.

**Conclusion:**

Although heterozygous *TTN*tv in the A‐band is known to cause dilated cardiomyopathy, we present evidence that these variants may in some cases cause a novel, dominant skeletal myopathy with a limb‐girdle pattern of weakness. These findings emphasize the importance of multidisciplinary care for patients with A‐band *TTN*tv who may be at risk for multisystem disease.

## Graphical abstract

Recent reports show pathogenic variants in *TTN* may result in a broader phenotypic spectrum than previously recognized. We investigated *TTN* genotype‐phenotype correlations in probands with skeletal myopathy and/or cardiomyopathy. Although heterozygous *TTN*tv in the A‐band are known to cause dilated cardiomyopathy, we present evidence that these variants may in some cases cause a novel, dominant skeletal myopathy with a limb‐girdle pattern of weakness.

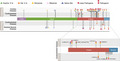

## 1 INTRODUCTION

The advent of massively parallel sequencing technologies in neuromuscular and cardiovascular clinics has transformed the diagnostic evaluation of skeletal myopathy and cardiomyopathy conditions. Genetic testing is often performed early in the diagnostic pathway and typically consists of complete sequencing of the coding regions of many genes, including those whose large size previously precluded routine testing, such as *TTN* (OMIM #188840). *TTN*, the gene for the muscle protein titin, is the largest human protein and has several vital functions within sarcomeres (Gautel, 2011; Granzier & Irving, 1995; Musa et al., 2006; Tskhovrebova & Trinick, 2010). Two titin filaments span the cardiac sarcomere; the terminal ends are embedded into the Z‐disk and the M‐band. The remainder of the protein is distributed between the A‐band and the flexible I‐band.

The sheer size of *TTN* has presented challenges for both sequencing techniques and interpretation of results. Complete sequencing of *TTN*, once impractical, costly, and rarely performed, is now routine in the evaluation of patients with skeletal myopathy and/or cardiomyopathy. However, the high frequency of variants in *TTN* (Akinrinade, Koskenvuo, & Alastalo, 2015), incomplete penetrance (Golbus et al., 2012; McNally, 2012), and limited understanding of the spectrum of disease, particularly neuromuscular disease, has led to uncertainties in determining the clinical significance of *TTN* variants, and many identified in clinical practice are dismissed as incidental.

Pathogenic variants in *TTN* are known to cause dilated cardiomyopathy (DCM; Herman et al., 2012) and several skeletal myopathy phenotypes. Dominant *TTN* truncating variants (*TTN*tv) in the A‐band are the leading genetic cause of DCM, accounting for up to 20‐30% of cases (Herman et al., 2012). Penetrance is age‐related and incomplete. Dominant *TTN*‐related DCM is not known to involve skeletal muscle weakness. Two dominant *TTN*‐related skeletal myopathies have been described; tibial muscular dystrophy (TMD), a dominant, distal myopathy (caused by 11‐base pair insertion/deletion (Hackman et al., 2002) as well as frameshift or nonsense variants in exons Mex5 and Mex6 (Hackman et al., 2008)) and hereditary myopathy with early respiratory failure (HMERF), a dominant distal‐onset myopathy caused by missense variants in exon 344 (Pfeffer et al., 2012). Neither is known to cause cardiomyopathy. Recessive skeletal myopathies associated with *TTN* include limb‐girdle muscular dystrophy type 2 J (LGMD2 J or LGMDR10; caused by the TMD‐related M‐band deletion in the homozygous state; Evila, 2014; Hackman et al., 2002), and centronuclear myopathy (caused by recessive truncating or in‐frame deletions/duplications; Ceyhan‐Birsoy et al., 2013). Recessive *TTN*tv have also been reported to cause several severe childhood‐onset phenotypes involving both skeletal muscle and cardiac disease, including early onset myopathy with fatal cardiomyopathy (Carmignac et al., 2007).

Commercial testing laboratories have variable methods and practices with respect to the detection, interpretation, and reporting of genetic variants (Shah et al., 2018; Yang et al., 2013), particularly for *TTN* (Bönnemann, 2018). Some laboratories report rare *TTN* missense variants as variants of uncertain significance (VUS), while others consider missense variants benign and do not report them. In addition, copy number variants are inconsistently detected and reported by diagnostic laboratories. Concurrent multidisciplinary evaluation of affected individuals in the same family may lead to discordant test interpretations, with a familial variant interpreted as likely pathogenic on a cardiomyopathy panel, but as a VUS in the same laboratory's neuromuscular panel. Finally, the clinical presentation of patients with pathogenic/likely pathogenic *TTN* variants affecting the A‐band of the protein may not match that described in the literature. Uncertainty regarding the clinical implications of those *TTN* variants presents challenges to clinicians undertaking genetic diagnosis and management of patients with neuromuscular and cardiac disease.

In this study, we investigated *TTN* variants, disease presentations, and transmission patterns in probands ascertained from neuromuscular and cardiovascular genetics clinics. For probands with atypical phenotypes, co‐segregation and phenotyping of family members were performed to characterize the intrafamilial spectrum of disease and mode of transmission.

## 2 METHODS

### 2.1 Ethical compliance

This work was approved by the Office of Responsible Research Practices at both The Ohio State University Wexner Medical Center and Stanford Medical Center.

Individuals with *TTN* variants were ascertained from The Ohio State University Wexner Medical Center Neuromuscular and Cardiovascular clinics (adult), the Stanford Center for Inherited Cardiovascular Disease (pediatric and adult) and the Stanford Neuromuscular Disorders Program (pediatric and adult) from 2014 to 2018. Genetic testing was conducted as part of routine clinical care and included multigene panels or exome sequencing. Indications for testing included LGMD, uncharacterized myopathy/muscular dystrophy, and cardiomyopathy. Those with a *TTN* variant classified as pathogenic, likely pathogenic, or VUS via CAP/CLIA‐certified clinical testing were included in the initial cohort (Figure S1). Family members of probands identified by cascade testing were excluded. Probands were also excluded if a pathogenic or likely pathogenic variant in a gene other than *TTN* was believed to better explain their cardiovascular and/or neuromuscular condition. Probands were excluded if they had secondary cardiomyopathy due to existing coronary artery disease or, in the case of one proband, because the reported cardiovascular presentation was attributed to cardiotoxicity due to methamphetamine and alcohol abuse. Probands with heterozygous missense VUS were not included. Previous research indicates that *TTN* missense variants are present in the general population at a frequency much higher than the frequency of idiopathic DCM and/or skeletal muscle disease (Akinrinade et al., 2019; Begay et al., 2015; Norton, 2013). As such, aside from the established pathogenic *TTN* missense variants (e.g., in exon 344 causing HMERF), many testing labs do not report them.

To ensure consistent mapping and classification of all ascertained variants, genomic coordinates for each variant were confirmed and mapped to one reference transcript (NM_001267550.2, Human Genome reference sequence 37). Variants were classified using the five‐tier system (pathogenic, likely pathogenic, uncertain, likely benign, benign) for grading evidence for pathogenicity as recommended by the American College of Medical Genetics (ACMG; Nykamp et al., 2017; Richards et al., 2015). To ensure consistent evidence‐based classification, each variant was considered in the context of current literature on *TTN* variation, and its role in disease was reviewed and implemented in a point‐based scoring system based on ACMG variant interpretation guidelines (Nykamp et al., 2017). In some cases, this resulted in a different classification of the variant compared to that provided by the laboratory.

Retrospective chart review compiled the reported neuromuscular and/or cardiovascular findings for all probands. Individuals were classified into one of the three clinical categories based on chart review; (a) neuromuscular, with documented skeletal muscle weakness and no known cardiac disease; (b) cardiovascular, with cardiac disease and no known skeletal muscle weakness; or (c) cardioskeletal, with both skeletal muscle weakness and cardiovascular disease. Family members of probands with cardioskeletal phenotypes were offered clinical evaluation and genetic testing.

For each proband in the study, a minimum group of three clinicians determined whether the documented phenotype was consistent with the expected phenotype based on the type and location of *TTN* variant(s) identified, referencing current literature including and the Online Mendelian Inheritance in Man database (Online Mendelian Inheritance in Man, OMIM®. McKusick‐Nathans Institute of Genetic Medicine, Johns Hopkins University [Baltimore, MD], World Wide Web URL: https://omim.org/) Phenotypic presentation was classified as typical (consistent with published literature), atypical (similar cases not published), or unknown in the case of VUS (Table S1). The determination of phenotypic concordance was not attempted for VUS because of the uncertainty of the variant to cause any specific phenotype.

Demographic and clinical characteristics were summarized using descriptive statistics. Comparisons between genotype and phenotype were assessed using descriptive statistics as well as Fisher's exact test. Comparison tests were limited to the highest pathogenic variant per proband. Pathogenic and likely pathogenic variants were combined when compared to VUS across phenotypes. Statistical analysis was performed using SAS/STAT statistical software (v9.4 SAS for Windows, SAS Institute Inc.).

## 3 RESULTS

### 3.1 Cohort ascertainment and demographics

Forty‐nine probands met criteria for inclusion in the study. The majority of the cohort (34/49; 69.4%) was ascertained from cardiovascular clinics and the remaining (15/49; 30.6%) from neuromuscular clinics. There were 29 males (59.2%) and 20 females (40.8%). Twelve probands (24.5%) had a pediatric onset of symptoms, while 37 probands (75.5%) had adult onset. The average age was 43.5 years (range 2‐80 years) at the time of chart review or death. Two probands (4.1%) were deceased at the time of chart review.

### 3.2 Phenotypic classification

Of the 49 probands included in the study, 30 (61.2%) were classified as having a cardiovascular phenotype and 12 (24.5%) as having a neuromuscular phenotype. Interestingly, seven (14.3%) probands had an atypical, cardioskeletal phenotype.

All probands in the cardiovascular cohort were identified in cardiovascular clinics; the majority were male (19/30, 63.3%) and had adult onset of symptoms (26/30, 86.6%). Similarly, all probands in the neuromuscular cohort were identified in neuromuscular clinics, 5/12 were male (41.6%), and 5/12 (41.6%) had pediatric onset of symptoms. In the cardioskeletal cohort, 4/7 (57.1%) were identified in cardiovascular clinics, 5/7 (71.4%) were male, and 4/7 (57.1%) had pediatric onset of symptoms (Table 1).

**Table 1:** Cardioskeletal summary data.

**Cardioskeletal Disease**										
**ID**	**Age** **Gender**	**Onset**	**Genotype**	**Band (Exon)**	**Reporting lab classification**	**Author classification**	**Neuromuscular phenotype (diagnosis)**	**Cardiac phenotype**	**Ascertain‐ment**	**Genotype/phenotype concordance**
1	77 M	Adult	c.79793 T>G (p.Leu26598*)	A (326)	P	LP	PUL, PLL (60y) (LGMD)	DCM (indeterminate etiology, CAD present), Afib (73 years)	Neuro	Atypical
2	35 F	Ped	c.92127dup (p.Pro30710Serfs*12)	A (338)	LP	LP	PUL, PLL (12y) (LGMD)	Peripartum cardiomyopathy, low‐normal EF (35 years)	Neuro	Atypical
3	60 M	Adult	c.47494C>T (p.Arg15832*)	A (253)	LP	P	PUL, muscle cramping and numbness (43y)	DCM, heart failure, conduction system disease (45 years)	Cardio	Atypical
4	29 F	Ped	c.80950G>T (p.Glu24416*)	A (326)	LP	LP	PLL, DLL (2y), fatty atrophy of paraspinal muscles	DCM (28 years), heart failure (28y)	Cardio	Atypical
5	57 M	Ped	c.96076_107488del	A/M (346‐362)	LP	LP	PUL, PLL, DUL, DLL, gait abnormality (8y) (LGMD)	DCM (42 years), heart failure (57 years)	Neuro	Atypical
6	57 M	Adult	c.76717C>T (p.Arg25573*)	A (326)	LP	LP	PUL, PLL, fasciculations, muscle cramping, muscle twitching, spontaneous abnormal muscle contraction (49y) (LGMD)	DCM (55 years)	Cardio	Atypical
7	17 M	Ped	c.47269+ 2T>C c.52307_52310dupTTGA (p.Glu17437Aspfs*2)	A (252) A (274)	LP LP	LP LP	PUL, DUL (12y)	RCM (14 years), s/p heart transplant	Cardio	Atypical

Note

All variants correspond to transcript NM_001267550.2. Protein effect provided when available. Ascertainment described the clinic (neuromuscular or cardiovascular) to which the proband originally presented.

Abbreviations: Afib, atrial fibrillation; CAD, coronary artery disease; DCM, dilated cardiomyopathy; DLL, distal lower limb weakness; F, female; LGMD, limb‐girdle muscular dystrophy; LP, likely pathogenic; M, male; NICM, non‐ischemic cardiomyopathy; P, pathogenic; Ped, pediatric onset; PLL, proximal lower limb weakness; PUL, proximal upper limb weakness, DUL, distal upper limb weakness; RCM, restrictive cardiomyopathy.

### 3.3 *TTN* variants

*TTN* variant data were compiled for all probands, including the variant classification (pathogenic, likely pathogenic, VUS), type (missense, frameshift, nonsense, splice site), and location by the sarcomeric domain (Z‐disc, I‐band, A‐band, M‐band; Table S1). Four probands have two *TTN* variants (4/49); three neuromuscular probands and one cardioskeletal proband. All other probands (45/49) were heterozygous for *TTN*tv.

In the cardiovascular cohort, the most frequent variant type was nonsense (10/30, 33.3%), followed by frameshift deletions (9/30, 26.6%) and frameshift insertion/duplications (6/30, 23.3%). The most frequent variant type identified in probands with a neuromuscular phenotype was splice site (7/15, 46.6%), followed by frameshift duplications, nonsense, and missense (each 2/15, 13.3%). Two neuromuscular probands have known pathogenic missense variants resulting in HMERF and demonstrate a typical HMERF phenotype. For the seven probands in the cardioskeletal cohort (one of whom had two *TTN* variants identified), four nonsense (57.1%), two frameshift duplications (28.6%), one frameshift deletion (14.3%) and one splice site variant (14.3%) was identified.

The highest proportion of heterozygous VUS were identified in probands in the neuromuscular cohort (3/9, 33.3%) followed by the cardiovascular cohort (6/30, 20.0%). No heterozygous *TTN* variants (0/6) were classified as VUS in the cardioskeletal cohort. Probands with two identified *TTN* variants were excluded from this analysis as we are unable to determine the individual impact of each variant. The proportion of pathogenic/likely pathogenic and VUS did not differ significantly between the three cohorts (Fisher's *p* = 0.33).

**Figure 1:**
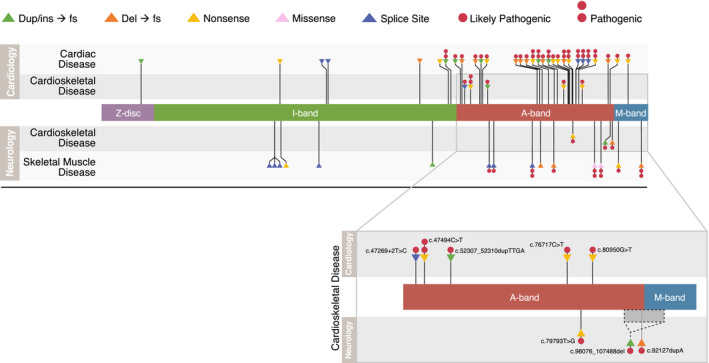
*TTN* variant sarcomere location by clinical category. The location of all *TTN* variants for each of the three symptom categories are shown. The titin gene is shown with sarcomeric bands represented by varying colors. Each point on the gene represents one *TTN* variant identified in our cohort. Variants above the gene are from individuals ascertained in cardiovascular clinics, variants below the gene are from individuals ascertained in neuromuscular clinics. The innermost variants are from cardioskeletal patients—the outermost variants are from the cardiac disease group and the skeletal muscle disease group. Inset shows the specific variant location for cardioskeletal probands. Dup/ins, duplication/insertion; fs, frameshift; del, deletion.

**Figure 2:**
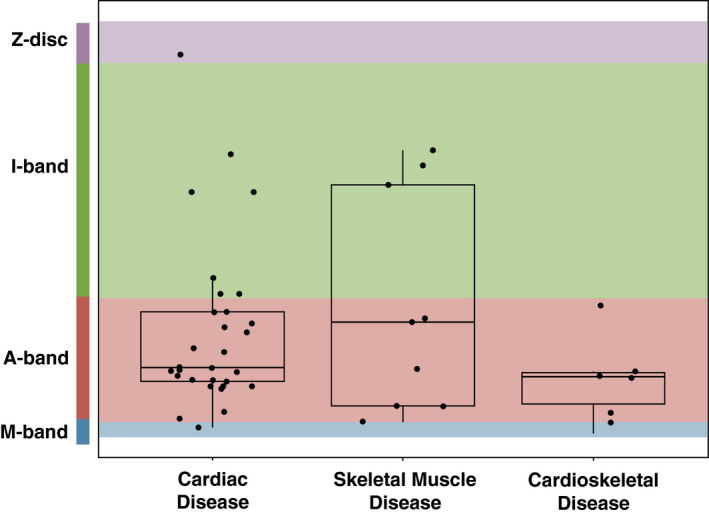
Distribution of *TTN* variants throughout the *TTN* gene by clinical category. Probands with multiple *TTN* variants were excluded from this figure.

In the cardioskeletal cohort, 100% of heterozygous variants (6/6) affected the A‐band, in contrast to 73.3% (22/30) of heterozygous variants in the cardiovascular cohort, and 55.6% (5/9) of heterozygous variants in the neuromuscular cohort (Fisher's *p* = 0.17). The majority of *TTN*tv in cardioskeletal probands were located in the distal end of the A‐band (Figure 1). The distribution of variants across all groups is shown in Figure 2.

Author re‐evaluation of all 53 *TTN* variants resulted in seven reclassifications from that provided by the reporting laboratory (7/53, 13.2%; Table S1). Four variants were downgraded (three pathogenic to likely pathogenic; one pathogenic to VUS) and three were upgraded (two likely pathogenic to pathogenic; one VUS to pathogenic).

### 3.4 Neuromuscular phenotypes

At the time of genetic testing, seven probands had an existing clinical diagnosis of uncharacterized myopathy/muscular dystrophy, five had an existing clinical diagnosis of uncharacterized congenital myopathy/muscular dystrophy and four had an existing clinical diagnosis of LGMD (Table S1). Two unrelated probands (one adult, one pediatric) had been clinically diagnosed with spinal muscular atrophy (with negative SMN1 testing) prior to undergoing additional genetic testing including *TTN*.

Various patterns of skeletal muscle weakness were identified in the neuromuscular and cardioskeletal cohorts, with proximal‐predominant and lower limb weakness most commonly reported (Figure S2). In the neuromuscular group, the most prevalent patterns of weakness were proximal lower limb weakness (reported in 91.7%), proximal upper limb weakness (83.3%), and distal lower limb (83.3%). In the cardioskeletal group, the most prevalent patterns of weakness were also proximal lower limb weakness (reported in 85.7%) and proximal upper limb weakness (71.4%). Five of seven probands in the cardioskeletal group (71.4%) had a history of muscle cramping.

Elevated creatine kinase (CK; >200 U/L) was identified in 5/12 neuromuscular probands (average CK 261.72 U/L [standard deviation 226], range 54‐826), and 3/7 cardioskeletal probands (average CK 1078 U/L [SD 2289], range 79‐6684). Electromyography studies were performed in eleven neuromuscular probands and three cardioskeletal probands; 10/11 neuromuscular probands had abnormal EMG results (nine myopathic, one neurogenic) and 2/3 cardioskeletal probands had abnormal EMG results (both myopathic).

### 3.5 Cardiovascular phenotypes

**Figure 3:**
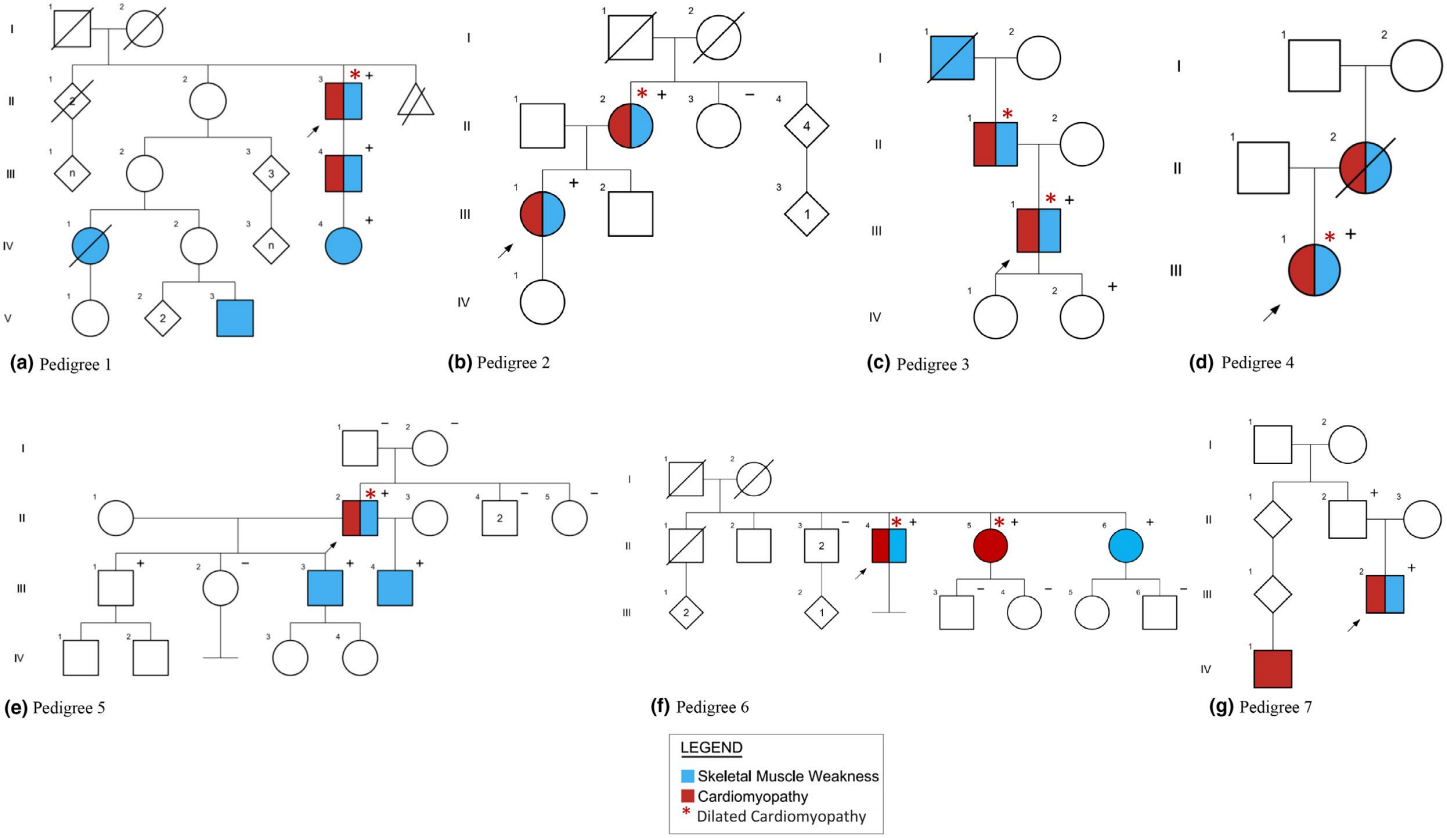
Pedigrees (a–g) of cardioskeletal probands.

Cardiomyopathy was present in 33/49 (67.3%) probands, and of these 28 (84.8%) met DCM criteria. For the 28 probands in our cohort who had DCM, 5 (17.8%) also had a neuromuscular phenotype, labeled as “cardioskeletal” (Figure 3). The majority of probands in both the cardiovascular and cardioskeletal groups had been diagnosed with heart failure (22/31 [71.0%] and 5/7 [71.4%], respectively).

Twenty‐one probands (42.9% of the cardiac group) had a history of arrhythmia. Atrial fibrillation (AF) was present in 4/49 probands (8.2%), three of whom had early onset AF (diagnosis <66 years). One proband (proband 43), diagnosed with an early onset arrhythmia, was treated with a pacemaker at age 6. This individual has a family history of early onset AF in her mother (*TTN*tv+) and history of DCM requiring heart transplant in her maternal uncle (*TTN*tv+).

### 3.6 Genotype‐phenotype concordance

**Figure 4:**
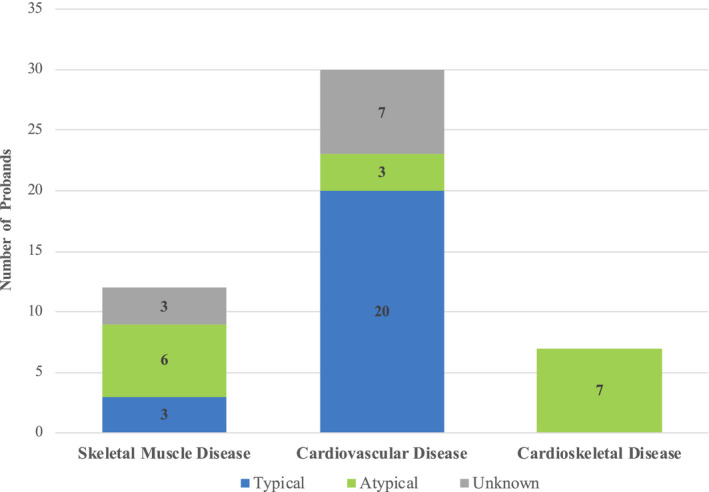
Genotype‐phenotype concordance. This histogram depicts the proportion of individuals within each clinical category whose phenotype was considered to be consistent with the literature‐reported genotype. For each proband in the study, a minimum group of three clinicians determined whether the documented phenotype was consistent with the expected phenotype based on the type and location of *TTN* variant(s) identified, referencing current literature. Probands were categorized as having a typical phenotype, an atypical phenotype, or unknown (in the case of VUS).

Among the cardiovascular cohort, 66.6% of patients had a phenotype that was expected for their genotype based on previous literature (“typical”), while 10% had a phenotype which was not supported by the literature (“atypical”) and 23.4% were unknown (in the case of VUS). Among the neuromuscular cohort, only 25% had typical phenotypes, 50% had atypical phenotypes, and 25% were unknown. Among the cardioskeletal cohort, no probands had phenotypes considered consistent with the identified genotype (100% atypical; Figure 4).

### 3.7 Probands with cardioskeletal phenotypes

All cardioskeletal probands (n = 7) had onset of neuromuscular weakness before diagnosis of cardiomyopathy. In general, these individuals had pediatric or young adult‐onset muscle weakness (average age of onset at 28 [2‐60]) followed by adult onset of cardiomyopathy (average age at cardiovascular diagnosis 44 [14‐73]). Variable patterns of neuromuscular weakness were documented, though most had proximal‐predominant and/or lower‐limb weakness. In childhood and young adulthood, the weakness did not consistently lead to medical evaluation, though it did impact the ability to perform daily tasks such as gym and physical activities, hair brushing, carrying infants, and in one case resulted in exclusion from military service. The oldest persons remained ambulatory, though in some cases with assistive devices. One proband died from heart failure at 59.

All cardioskeletal probands had a family history of cardiac disease, skeletal muscle disease, or both (Figure 3). Five of seven pedigrees demonstrated apparently dominant transmission. In each family available for cascade testing, at least one affected family member was also identified to share the proband's *TTNtv*. Family Studies are summarized below. All variants correspond to transcript NM_001267550.2 unless otherwise specified, protein effect provided when available.

#### 3.7.1 Pedigree 1

The proband (proband 1; II‐3, Figure 3a) was diagnosed with LGMD at 60 and subsequently DCM at 73. Genetic testing revealed a likely pathogenic A‐band *TTN*tv on neuromuscular panel testing (c.79793 T>G, p.Leu26598*). His son (III‐4) developed “tachycardia‐induced cardiomyopathy” at 38, began experiencing upper extremity weakness at 39, and shares the *TTN*tv identified in his father. His granddaughter through this son (IV‐4) also shares the same variant and has both skeletal muscle weakness and tachycardia. The proband also has a great niece (IV‐1) who died at 25 from uncharacterized muscular dystrophy (no information on cardiac status), as well as a great nephew once removed (V‐3) with an uncharacterized form of muscular dystrophy and no cardiac involvement. Genetic testing of these individuals was not performed.

#### 3.7.2 Pedigree 2

The proband (proband 2; III‐1, Figure 3b) experienced proximal‐predominant weakness in childhood and was diagnosed with LGMD in her 20 s. Neuromuscular panel testing identified a duplication in the A‐band of *TTN*, (c.92127dup, p.Pro30710Serfs*12), classified as a VUS. The proband's mother (II‐2) had an “enlarged heart,” was referred for cardiogenetic evaluation and found to have DCM (at 59) and reported a history of muscle cramping and frequent falls. Cardiomyopathy panel testing via the same laboratory identified the same *TTN* variant as the proband, although on this test the variant was classified as likely pathogenic. The proband subsequently developed peripartum cardiomyopathy with low‐normal ejection fraction (EF).

#### 3.7.3 Pedigree 3

The proband (proband 3; III‐1, Figure 3c) developed proximal upper‐limb weakness and muscle cramping at 43 and was diagnosed with DCM, heart failure, and conduction system disease at 45. Cardiomyopathy panel testing revealed an A‐band *TTN*tv (c.47494C>T, p.Arg15832*), interpreted as pathogenic. His father (II‐1) was similarly affected by DCM and muscle weakness but has not had genetic testing. One daughter of the proband tested positive for the same variant but cardiac examination at 30 years old did not show signs of disease. Family history also included a paternal grandfather (I‐1) with muscle weakness, though this individual was not available for genetic testing.

#### 3.7.4 Pedigree 4

The proband (proband 4; III‐1, Figure 3d) had proximal and distal lower‐limb weakness since childhood as well as fatty atrophy of the paraspinal muscles. She was diagnosed with DCM and heart failure at 28. A A‐band *TTN*tv (c.80950G>T, p.Glu24416*), classified as likely pathogenic, was identified on cardiomyopathy panel testing. The proband's mother (II‐2) reportedly experienced difficulty walking and frequent falls throughout her life and died at 50 from heart failure before genetic testing could occur.

#### 3.7.5 Pedigree 5

The proband (proband 5; II‐2, Figure 3e) reported muscle weakness since childhood, with difficulty running and lifting, and eventually developed a unique gait marked by profound external rotation of bilateral hips. He was diagnosed with DCM and subsequent heart failure at 42 years old. A 16.430 kb heterozygous deletion spanning part of the A‐ and M‐bands of *TTN* (c.96076_107488del), classified as likely pathogenic, was identified via neuromuscular panel testing; the deletion had not been identified on a previous neuromuscular panel which also included *TTN* sequencing. The deletion is *de novo*; it was also identified in two symptomatic sons. One son (III‐4) had childhood‐onset of muscle weakness (proximal>distal) and the other son (III‐3) had adult‐onset of muscle weakness and a borderline‐low EF. This pedigree has been previously reported in detail (Roggenbuck et al., 2019).

#### 3.7.6 Pedigree 6

The proband (proband 6; II‐5, Figure 3f) developed proximal‐predominant muscle weakness, fasciculations, and cramping at 49, leading to a diagnosis of LGMD. He was later diagnosed with DCM at 55. A cardiomyopathy panel identified a *TTN* truncating A‐band variant (c.76717C>T; p.Arg25573*), interpreted as likely pathogenic. Two of the proband's sisters were also identified to have this *TTN* variant, one of whom was diagnosed with DCM at 38 (II‐5), the other who developed muscle weakness in late middle age (II‐6).

#### 3.7.7 Pedigree 7

The proband (proband 7; III‐2, Figure 3g) experienced proximal and distal upper limb weakness at 12 and was diagnosed with restrictive cardiomyopathy (RCM) at 14, requiring heart transplant. Two *TTN* variants (c.47269+2 T>C and c.52307_52310dupTTGA, p.Glu17437Aspfs*2), interpreted as likely pathogenic and pathogenic, respectively, were identified on cardiomyopathy panel testing. This proband has a paternal first cousin once removed (IV‐1) who also had RCM requiring heart transplant at a young age, though this individual was unavailable for genetic testing.

Although the phenotypes presented above have not been widely reported to result from *TTN* variants, the type of variant (i.e., *TTN*tv in the A‐band as seen in all pedigrees) and/or the segregation of the variant with a cardioskeletal phenotype (pedigrees 1, 2, and 5) are suggestive of pathogenicity.

## 4 DISCUSSION

Our identification of several probands with atypical cardioskeletal phenotypes and heterozygous pathogenic/likely pathogenic variants suggests the spectrum of *TTN*‐related disease may be broader than currently recognized. We provide evidence of a novel dominant cardioskeletal phenotype associated with heterozygous *TTN*tv affecting the distal A‐band, characterized by proximal‐predominant muscle weakness and DCM, identified in five probands (10.2% of the cohort). *TTN*‐related DCM is inherited in an autosomal dominant fashion but is not known to have extracardiac manifestations. *TTN*‐related neuromuscular diseases (other than TMD and HMERF) are considered to be recessive. Several recent reports emphasize that heterozygosity for *TTN*tvs is insufficient to establish a diagnosis of a neuromuscular titinopathy without mRNA and protein studies (Savarese et al., 2018). Cardioskeletal phenotypes associated with *TTN* have been reported in several case series, all describing recessive congenital myopathies with variable cardiovascular involvement (Carmignac et al., 2007; Chauveau et al., 2014; Oates et al., 2018a, 2018b). Other authors have alluded to multisystem phenotypes (mild cardiomyopathy and skeletal myopathy) in carriers of A‐band *TTN* variants (Ceyhan‐Birsoy et al., 2013), and identified relatives of probands with possible multisystem involvement (Oates et al., 2018a, 2018b).

Our data illustrate that *TTN*tv of established or potential clinical significance is identified in routine clinical practice in both cardiovascular and neuromuscular settings. However, the clinical impact of these variants in the context of the proband's specific presentation is frequently unclear, particularly in neuromuscular clinics. The majority of pathogenic *TTN* variants ascertained in cardiovascular probands (66.6%) were associated with the expected phenotype of autosomal dominant DCM, whereas only 25% of pathogenic *TTN* variants identified in neuromuscular probands corresponded with the phenotype reported in the literature. The observed genotype/phenotype discordance highlights the challenges in interpreting *TTN* variants in the clinic, particularly in the context of neuromuscular genetic evaluations. Clinical expertise and ongoing consideration of new evidence remain essential in the approach to *TTN* variants.

*TTN* variants are currently known to cause several neuromuscular diseases, including TMD (distal myopathy), LGMD2 J (an early onset, proximal myopathy; Hackman et al., 2008), HMERF (muscle weakness progressing from distal lower limbs to generalized weakness; Pfeffer et al., 2012), and centronuclear myopathy (recessive congenital myopathy; Ceyhan‐Birsoy et al., 2013) among others. Given their genotypes and presentations, the majority of probands in our study with skeletal myopathy did not fit within any of these well‐established diagnostic entities. Our clinical data suggest that in addition to DCM, heterozygous *TTN*tv may also cause dominant skeletal myopathy in some individuals. Dominant transmission of this phenotype may have previously escaped notice given that *TTN*tv has incomplete or age‐dependent penetrance, as seen in *TTN*‐related DCM (Morales & Hershberger, 2013), and younger, at‐risk relatives may be asymptomatic, impairing the ability to characterize inheritance patterns. Importantly, our cohort is generally older than those described in previous reports of skeletal titinopathies (which overwhelmingly describe congenital myopathies). In addition, these data add to the emerging question of whether *TTN* may be associated with cardiac phenotypes other than DCM, including AF and RCM.

Five of seven cardioskeletal pedigrees support the dominant transmission of skeletal muscle disease, cardiovascular disease and/or cardioskeletal disease (Figure 3). In the majority of families where cascade testing was performed, the *TTN*tv was identified in at least one affected family member, and in one case was shown to be *de novo* (Figure 3e), supporting a dominant mechanism. ACMG criteria consider this strong evidence for pathogenicity (Richards et al., 2015). Further, all cardioskeletal probands were identified to have heterozygous *TTN*tv affecting the A‐band, the same type of *TTN*tv commonly observed in autosomal dominant DCM. For this reason, the cardioskeletal presentation could be seen as an expansion of the typical *TTN*‐related DCM phenotype, or a distinct entity which appears to share similar genetic etiology. In favor of the second interpretation, a family history of skeletal muscle weakness was observed in 5/7 cardioskeletal probands, suggesting that the multisystem presentation clusters in families.

It is not known why *TTN*tv in the A‐band can cause isolated DCM in most families and may cause a cardioskeletal phenotype in others. One possible explanation is that most *TTN*tv in our cardioskeletal probands falls in the distal end of the A‐band, which include constitutively expressed exons common to both skeletal muscle isoforms (N2A) and cardiovascular isoforms (N2B, N2BA, fetal isoforms; Prado et al., 2005). It is reasonable to hypothesize that variants that impact an exon expressed in both skeletal and cardiac muscle could lead to multisystem involvement. Other explanations are possible, including not yet described genetic and/or environmental modulators of *TTN* expression.

Almost half (46.6%) of the *TTN*tvs in the neuromuscular cohort were classified as VUS, compared with 20% in the cardiovascular cohort and none in the cardioskeletal cohort. Most variants interpreted as pathogenic or likely pathogenic were clustered in the A‐band of *TTN*, whereas variants identified in the neuromuscular group were distributed more widely across the protein. Variants outside the A‐band are categorically less likely to be meet ACMG criteria for pathogenicity, given the established clinical significance of A‐band variants supported by multiple lines of clinical and functional evidence (LeWinter & Granzier, 2013; Oates et al., 2018a, 2018b).

### 4.1 Limitations

The retrospective, clinic‐based nature of the study did not allow for testing of a matched control population. *TTN*tv are known to occur in the general population, though the frequency is lower than initially reported (Akinrinade et al., 2015) and evidence supports reduced penetrance (Norton, 2013). Most *TTN*tv present in the general population impact exons not expressed in functional isoforms and therefore are not expected to cause disease (Ware & Cook, 2018).

Missense variants not predicted to truncate the protein (or not known to cause disease, i.e., HMERF; Pfeffer et al., 2012) were excluded from analysis in this study. *TTN* missense changes are largely considered to be tolerated (Herman et al., 2012; Roberts et al., 2015), however, further study is needed to investigate their potential impact.

This study characterized phenotypes and genotypes of probands with *TTN* variants ascertained in both cardiovascular and neuromuscular clinics. Functional analysis to understand mechanisms of variable expressivity in *TTN*tvs is a subject of future study.

## 5 CONCLUSIONS

The widespread application of panel testing in cardiovascular and neuromuscular clinics has led to the identification of *TTN*tvs in both patient populations. However, interpreting the clinical significance of these variants is challenging, particularly for patients with skeletal myopathy. Our data suggest the spectrum of *TTN*‐related disease may be broader than currently recognized.

We provide evidence that distal A‐band *TTN*tv, typically associated with autosomal dominant DCM, may also cause a previously unrecognized autosomal dominant cardioskeletal phenotype in some families. Dominant variants in *TTN* may underlie a proportion of skeletal myopathy disorders which currently remain genetically uncharacterized and should be considered in the differential diagnosis of patients with skeletal muscle weakness and cardiomyopathy. Further study is needed to characterize the spectrum of *TTN*‐related disease, as well as the underlying genetic mechanisms. We anticipate that the clinical and genetic characterization of novel titinopathies will facilitate variant interpretation and lead to improved diagnostic yields.

## CONFLICT OF INTERESTS

C. Tan, M. Vatta, T. Winder, and A. Morales are employees of Invitae Corporation. B. Elsheikh is a medical consultant for Biogen Inc.

## AUTHORS CONTRIBUTIONS

Conceptualization: KAR, AM, JR. Data collection: KAR, TM, CS, CAT, LV, BT, AM, JR. Data analysis: KAR, GB, MP. Data interpretation: KAR, TM, CS, MV, TLW, BE, REH, JTK, AM, JR. Manuscript writing: KAR, TM, CS, CAT, MV, TLW, REH, JTK, AM, JR.

## Supporting information

Supplementary Material

## Data Availability

Variant data are publicly available on ClinVar. Additional data are available upon request.
