# Supplementary material for: Novel heterozygous truncating titin variants affecting the A‐band are associated with cardiomyopathy and myopathy/muscular dystrophy
Source: Mol Genet Genomic Med. 2020 Aug 20;8(10):e1460. doi: 10.1002/mgg3.1460 (PMC7549586; doi:10.1002/mgg3.1460)
Supplement: Supplementary file 1 — Supplementary Material [file MGG3-8-e1460-s001.docx]

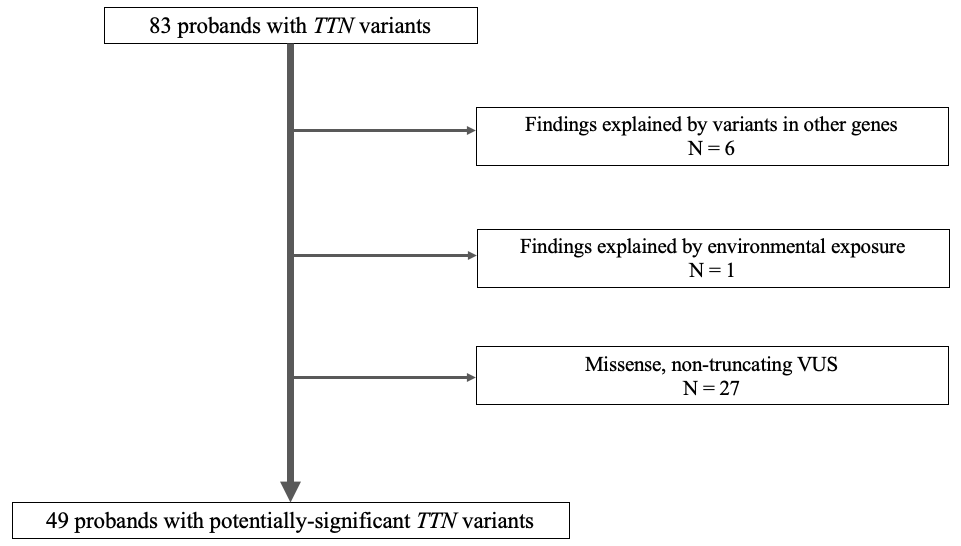


**Figure S1. Inclusion/Exclusion Criteria** Abbreviations: VUS, variants of unknown significance


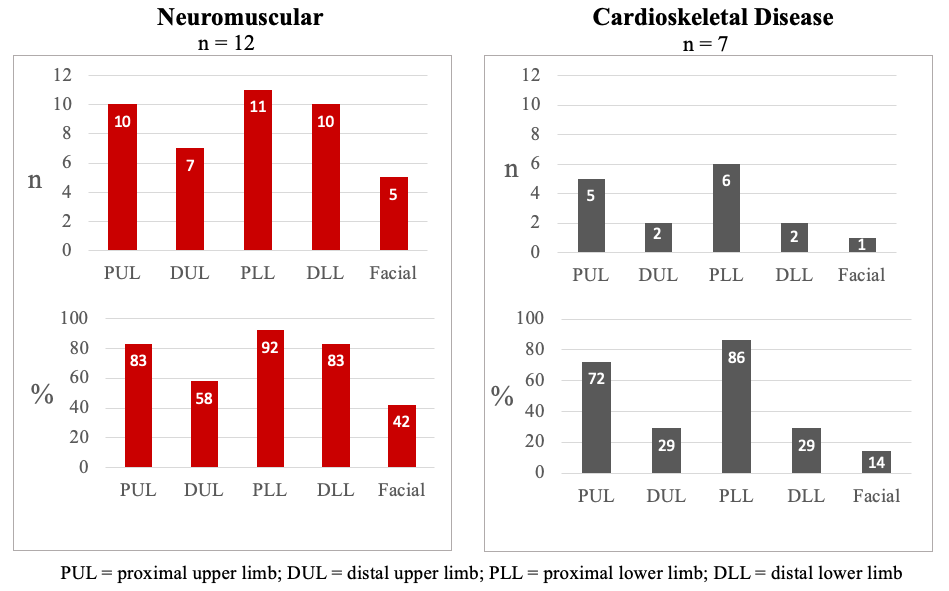


**Figure S2. Weakness Patterns in Neuromuscular and Cardioskeletal Disease** Abbreviations: PUL, proximal upper limb; DUL, distal upper limb; PLL, proximal lower limb; DLL, distal lower limb

Table S1: Cohort Summary Data

| **Cardioskeletal Disease** | | | | | | | | |
| --- | --- | --- | --- | --- | --- | --- | --- | --- |
| **ID** | **Genotype** | **Band** | **Reporting Lab Classification** | **Author Classification** | **Neuromuscular Phenotype (Diagnosis)** | **Cardiac phenotype** | **Ascertain-ment** | **Genotype/Phenotype concordance** |
| 1 | c.79793T>G (p.Leu26598*) | A | P | LP | PUL, PLL (60y)  (LGMD) | DCM (indeterminate etiology, CAD present), Afib (73y) | Neuro | Atypical |
| 2 | c.92127dup (p.Pro30710Serfs*12) | A | LP | LP | PUL, PLL (12y)  (LGMD) | Peripartum cardiomyopathy, low-normal EF (35y) | Neuro | Atypical |
| 3 | c.47494C>T (p.Arg15832*) | A | LP | P | PUL, muscle cramping and numbness (43y) | DCM, heart failure, conduction system disease (45y) | Cardio | Atypical |
| 4 | c.80950G>T (p.Glu24416*) | A | LP | LP | PLL, DLL (2y), fatty atrophy of paraspinal muscles | DCM (28y), heart failure (28y) | Cardio | Atypical |
| 5 | c.96076_107488del | A/M | LP | LP | PUL, PLL, DUL, DLL, gait abnormality (8y)  (LGMD) | DCM (42y), heart failure (57y) | Neuro | Atypical |
| 6 | c.76717C>T (p.Arg25573*****) | A | LP | LP | PUL, PLL, fasciculations, muscle cramping, muscle twitching, spontaneous abnormal muscle contraction (49y)  (LGMD) | DCM (55y) | Cardio | Atypical |
| 7 | c.47269+2T>C  c.52307_52310dupTTGA  (p.Glu17437Aspfs*2) | A  A | LP  LP | LP  LP | PUL, DUL (12y) | RCM (14y), s/p heart transplant | Cardio | Atypical |
| **Neuromuscular Disease** | | | | | | | | |
| **ID** | **Genotype** | **Band** | **Reporting Lab Classification** | **Author Classification** | **Neuromuscular Phenotype (Diagnosis)** | **Cardiac phenotype** | **Ascertain-ment** | **Genotype/Phenotype concordance** |
| 8 | c.25063+1G>A | I | VUS | VUS | PUL. DUL, PLL, DLL (Myotubular myopathy) |  | Neuro | Atypical |
| 9 | c.29024C>A (p.Ser9675*) | I | VUS | VUS | PUL, PLL, FW  (LGMD) | Afib | Neuro | Atypical |
| 10 | c.31426+1G>C | I | VUS | VUS | PUL, PLL, EMG showed denervating process |  | Neuro | Unknown |
| 11 | c.100825C>T (p.Arg33609*) | M | P | LP | DUL, DLL, foot drop  (*TTN*-related myopathy) |  | Neuro | Atypical |
| 12 | c.95126C>G (p.Pro31709Arg) | A | P | P | HMERF |  | Neuro | Typical |
| 13 | c.95187G>C (p.Trp31729Cys) | A | P | P | HMERF | Mild mitral valve regurgitation | Neuro | Typical |
| 14 | c.66160+2T>C  c.107460_107463dup, p.Gln35822Serfs*21 | A  M | LP  VUS | P  P | PUL, PLL, DLL, FW (LGMD) |  | Neuro | Typical |
| 15 | c.25063+1G>A  c.25063+1G>A | I  I | VUS  VUS | VUS  VUS | PUL, DUL, PLL, DLL, FW (Centronuclear myopathy), Biopsy: multiminicores | Mild cardiomegaly | Neuro | Unknown |
| 16 | c.42054dup (p.Arg14019fs)  c.69937_69939del (p.Asn23313del) | I  A | P  VUS | VUS  VUS | PUL, DUL, PLL, DLL  (Congenital myopathy) | Sinus arrhythmia | Neuro | Unknown |
| 17 | c.54190+1G>A | A | LP | LP | PUL, DUL, PLL, DLL, FW,  (Congenital myopathy) |  | Neuro | Atypical |
| 18 | c.75138_75141del | A | LP | LP | PUL, PLL, fatigue  (Stable congenital myopathy) |  | Neuro | Atypical |
| 19 | c.54812-1G>T | A | LP | LP | PUL, PLL, DUL, DLL, congenital elbow contractures  (Congenital myopathy) |  | Neuro | Atypical |
| **Cardiovascular Disease** | | | | | | | | |
| **ID** | **Genotype** | **Band** | **Reporting Lab Classification** | **Author Classification** | **Neuromuscular Phenotype (Diagnosis)** | **Cardiac phenotype** | **Ascertain-ment** | **Genotype/Phenotype concordance** |
| 20 | c.74045del (p.Gly24682fs) | A | LP | LP | Generalized weakness | DCM, heart failure | Cardio | Typical |
| 21 | c.74338C>T (p.Arg24780*) | A | P | P |  | DCM, heart failure | Cardio | Typical |
| 22 | c.97395del (p.Glu32466fs) | A | LP | LP | Foot numbness and paresthesia, lower limb hair loss and loss of pin sensation | DCM, heart failure, nonsustained ventricular tachycardia | Cardio | Typical |
| 23 | c.82273C>T (p.Gln27425*) | A | LP | LP |  | DCM, heart failure | Cardio | Typical |
| 24 | c.57769C>T (p.Arg19257*) | A | LP | LP |  | DCM, heart failure | Cardio | Typical |
| 25 | c.31763-1G>A | I | VUS | VUS | Muscle aches and pains, fibromyalgia | Premature ventricular contraction | Cardio | Unknown |
| 26 | c.86821+2T>A | A | P | P | Generalized weakness | DCM, heart failure, arrhythmia | Cardio | Typical |
| 27 | c.3351dup, (p.Ser1118Ilefs*21) | Z | VUS | VUS |  | Non-ischemic cardiomyopathy, Afib | Cardio | Unknown |
| 28 | c.86821+2T>A | A | P | P |  | DCM, heart failure, ventricular tachycardia | Cardio | Typical |
| 29 | c.55460_55461del (p.Lys18487fs) | A | LP | LP |  | DCM, heart failure, conduction system disease | Cardio | Typical |
| 30 | c.83515C>T (p.Arg27839*) | A | P | P |  | DCM, heart failure | Cardio | Typical |
| 31 | c.76115dup (p.Asn25372fs) | A | P | P |  | DCM, LVNC, heart failure | Cardio | Typical |
| 32 | c.86821+2T>A | A | P | P |  | Non-dilated, nonischemic cardiomyopathy | Cardio | Typical |
| 33 | c.45316_45320dup (p.Arg15108fs) | I | P | P |  | Borderline EF, prominent arrhythmia | Cardio | Unknown |
| 34 | c.26028G>A (p.Trp8676*) | I | VUS | VUS |  | DCM, heart failure, Afib | Cardio | Unknown |
| 35 | c.79603C>T (p.Gln26535*) | A | P | LP | Multiple sclerosis | DCM, heart failure, arrhythmia | Cardio | Typical |
| 36 | c.87559G>T (p.Glu29187*) | A | P | P |  | DCM, arrhythmia, conduction system disease | Cardio | Typical |
| 37 | c.99496G>T (p.Glu33166*) | A | LP | LP | Facial weakness following TIA | DCM, heart failure, arrhythmia, conduction system disease | Cardio | Typical |
| 38 | c.64688del (p.Pro21563fs) | A | LP | LP | Motor weakness at birth | DCM, heart transplant 3y | Cardio | Typical |
| 39 | c.52063_52064insAlu | A | LP | LP |  | DCM, heart failure, arrhythmia, conduction system disease | Cardio | Typical |
| 40 | c.77100dup (p.Pro25701fs) | A | P | P |  | DCM, heart failure, arrhythmia, conduction system disease | Cardio | Typical |
| 41 | c.82658del (p.Gly27553fs) | A | P | P |  | DCM, arrhythmia, LA fascicular block, high PVC burden (15%) | Cardio | Typical |
| 42 | c.76507del, (p.Glu23862Lysfs*3) | A | VUS | LP |  | DCM, arrhythmia | Cardio | Typical |
| 43 | c.66039del (p.Ser22014fs) | A | LP | LP |  | Conduction system disease, mild systolic dysfunction, early onset arrhythmia, pacemaker 6y | Cardio | Atypical |
| 44 | c.45307C>T (p.Arg15103*) | I | VUS | VUS |  | Arrhythmia (11y), cardiac MRI showed mild bilateral ventricular dilation but no loss of function | Cardio | Unknown |
| 45 | c.30895+1G>A | I | VUS | VUS |  | Arrhythmia, conduction system disease (11y) | Cardio | Unknown |
| 46 | c.43727_43728del (p.Glu14576fs) | I | VUS | VUS | Mild weakness following stroke | DCM, heart failure, arrythmia, LBBB | Cardio | Unknown |
| 47 | c.56732dup (p.Asp18911fs) | A | LP | LP |  | DCM, conduction system disease (LA fascicular block) | Cardio | Typical |
| 48 | c.52077_52078delinsT (p.Lys17359fs) | A | LP | LP |  | Arrhythmia | Cardio | Atypical |
| 49 | c.104771C>A (p.Ser34924*) | M | LP | LP |  | DCM, heart failure, Afib | Cardio | Atypical |

Note: All variants correspond to transcript NM_001267550.2. Protein effect provided when available. Ascertainment described the clinic (neuromuscular or cardiovascular) to which the proband originally presented.

Abbreviations: LP, likely pathogenic; P, pathogenic; VUS, variant of unknown significance; PUL, proximal upper limb weakness, DUL, distal upper limb weakness; PLL, proximal lower limb weakness; DLL, distal lower limb weakness; LGMD, limb-girdle muscular dystrophy; HMERF, hereditary myopathy with early respiratory failure; FW, facial weakness; DCM, dilated cardiomyopathy; Afib, atrial fibrillation; CAD, coronary artery disease; NICM, non-ischemic cardiomyopathy; RCM, restrictive cardiomyopathy; TIA, transient ischemic attack; PVC premature ventricular contraction; LVNC, left ventricular non-compaction; LBBB, left bundle branch block.
